# Medication safety and chronic kidney disease in older adults prescribed metformin: a cross-sectional analysis

**DOI:** 10.1186/1471-2369-15-86

**Published:** 2014-06-07

**Authors:** Deborah L Huang, Itamar B Abrass, Bessie A Young

**Affiliations:** 1 Division of General Internal Medicine, University of Washington, Box 354765, 4245 Roosevelt Way NE, Seattle, WA 98105, USA; 2 Division of Gerontology and Geriatric Medicine, University of Washington, Seattle, WA, USA; 3 Epidemiology Research and Information Center, Health Services Research and Development Center for Innovation for Veteran-Centered and Value-Driven Care, Veterans Affairs Puget Sound Health Care System, Seattle, WA, USA; 4 Division of Nephrology, Kidney Research Institute, University of Washington, Seattle, WA, USA

**Keywords:** Aged, Chronic kidney disease, Diabetes mellitus, Drug prescriptions, Medication safety, Metformin, Renal insufficiency

## Abstract

**Background:**

Medication safety in patients with chronic kidney disease (CKD) is a growing concern. This is particularly relevant in older adults due to underlying CKD. Metformin use is contraindicated in patients with abnormal kidney function; however, many patients are potentially prescribed metformin inappropriately. We evaluated the prevalence of CKD among older adults prescribed metformin for type 2 diabetes mellitus using available equations to estimate kidney function and examined demographic characteristics of patients who were potentially inappropriately prescribed metformin.

**Methods:**

We conducted a cross-sectional analysis of older adults aged ≥65 years prescribed metformin from March 2008-March 2009 at an urban tertiary-care facility in Seattle, Washington, USA. CKD was defined using National Kidney Foundation-Kidney Disease Outcomes Quality Initiative criteria. Creatinine clearance was calculated using the Cockcroft-Gault equation; estimated glomerular filtration rate was calculated using the abbreviated Modification of Diet in Renal Disease (MDRD) and CKD-Epidemiology (EPI) Collaboration equations. Regression analyses were used to determine the associations between demographic characteristics and prevalent CKD.

**Results:**

Among 356 subjects (median age 69 years, 52.5% female, 39.4% non-Hispanic black), prevalence of stage 3 or greater CKD calculated by any of the equations was 31.4%. The Cockcroft-Gault equation identified more subjects as having CKD (23.7%) than the abbreviated MDRD (21.1%) or CKD-EPI (21.7%) equations (*P* < 0.001). Older age (OR = 1.13, 95% CI 1.08-1.19) and female sex (OR = 2.51, 95% CI 1.44-4.38) were associated with increased odds of potentially inappropriate metformin prescription due to CKD; non-Hispanic black race was associated with decreased odds of potentially inappropriate metformin prescription due to CKD (OR = 0.41, 95% CI 0.23-0.71).

**Conclusions:**

CKD is common in older adults prescribed metformin for type 2 diabetes, raising concern for potentially inappropriate medication use. No single equation to estimate kidney function may accurately identify CKD in this population. Medication safety deserves greater consideration among elderly patients due to the widespread prevalence of CKD.

## Background

Medication safety in older adults is a growing concern, particularly in those with chronic kidney disease (CKD). Although new CKD estimating equations such as the abbreviated Modification of Diet in Renal Disease (MDRD) Study equation [1] and Chronic Kidney Disease Epidemiology Collaboration (CKD-EPI) formula [2] are available, pharmacists tasked with the delivery of medications tend to use the older Cockcroft-Gault formula [3] to estimate CKD according to pharmaceutical manufacturer recommendations [4]. Metformin is an effective medication commonly prescribed to treat type 2 diabetes mellitus, but its use is potentially limited in adults ≥65 years of age due to increased prevalence of CKD [5,6]. The American Geriatrics Society recently recommended using estimated glomerular filtration rate (eGFR) to guide metformin use in older adults with diabetes: metformin should not be used when eGFR < 30 mL/min/1.73 m^2^, and lower dosages are recommended for eGFR 30–60 mL/min/1.73 m^2^, with more frequent kidney function monitoring [7]. Current U.S. drug labeling includes “renal impairment” (defined as “serum creatinine greater than or equal to 1.5 mg/dL [133 μmol/L] [males] or 1.4 mg/dL [124 μmol/L] [females] or an abnormal creatinine clearance”) as a contraindication to using metformin [8]. Additional precautions for older adults recommend “conservative” dosing, and to only start metformin in adults ≥80 years of age if creatinine clearance is normal [8,9]. A black box warning was issued by the U.S. Food and Drug Administration regarding the increased risk of metformin-associated lactic acidosis with older age as well as impaired kidney function [8], though metformin-associated lactic acidosis is a rare complication [10]. Thus, older adults are at higher risk of developing adverse events when using metformin due to underlying impaired kidney function.

The National Kidney Foundation (NKF) defines CKD as “either kidney damage [defined as pathologic abnormalities or markers of damage including abnormal blood, urine or imaging tests] or GFR [glomerular filtration rate] <60 mL/min/1.73 m^2^ for ≥3 months” [11]. Current available methods used in clinical practice to estimate GFR include: 1) calculating creatinine clearance using the Cockcroft-Gault equation; 2) calculating eGFR using the abbreviated MDRD equation; or 3) the CKD-EPI formula. These equations may not accurately estimate kidney function for older adults [12,13] and are reported to give discordant results among older adults with an eGFR ≥60 mL/min/1.73 m^2^[13-17]. Prescribing clinicians and clinical pharmacists may use different equations to estimate kidney function [4], which may lead to conflicting opinions regarding appropriate use of metformin due to disparities in estimated kidney function. Additionally, there are no uniform U.S. clinical practice guidelines for metformin use in kidney impairment and considerable variation in recommendations worldwide [18].

Few studies describe the prevalence of CKD in older adults (≥65 years of age) with diabetes mellitus, particularly among those taking metformin. Reported prevalence of CKD among U.S. older adults ranges from 25.9-44% [5,6,19]. A UK study reported 49% of adults ≥70 years old with diabetes mellitus had CKD stage 3 or higher [20]. Improved recognition of CKD in older adults with diabetes mellitus is needed to identify patients at increased risk of serious adverse events and to determine appropriate treatment plans. We hypothesized that older adults with CKD were at risk for potentially inappropriate treatment with metformin. The current study reports the prevalence of CKD among older adults with type 2 diabetes mellitus prescribed metformin at an urban tertiary-care facility and compares the prevalence of CKD using the Cockcroft-Gault, abbreviated MDRD and CKD-EPI equations to estimate kidney function.

## Methods

### Study subjects

Outpatient pharmacy records were reviewed to identify subjects meeting study inclusion criteria: aged ≥65 years who filled a prescription for metformin at any of the Harborview Medical Center (Seattle, Washington) outpatient pharmacies from March 1, 2008 to March 1, 2009. The diagnosis of type 2 diabetes mellitus was assumed for older adults prescribed metformin; older adults are unlikely to be taking metformin for off-label conditions. Three hundred fifty-six subjects were identified who met study inclusion criteria. The Harborview Medical Center outpatient pharmacy fills approximately 80% of prescriptions ordered at the facility for both outpatients and inpatients upon hospital discharge; the remainder of prescriptions are filled at outside pharmacies. The initial metformin prescription filled was used for subjects who obtained multiple metformin prescriptions during the study period. This study was approved by the IRB at the University of Washington (University of Washington Human Subjects Divison).

### Data collection

Age, sex, race/ethnicity, weight, serum creatinine, reported eGFR, prescribing provider and primary care provider were abstracted from the electronic medical record. Access to the electronic medical records used for this study is not freely available; permission to access these records was granted by Harborview Medical Center after study approval by the University of Washington Human Subjects Division. The most recent reported values for weight, serum creatinine and eGFR prior to the earliest metformin prescription were used to estimate kidney function. Serum creatinine values were generally obtained in the 12 months prior to the earliest metformin prescription fill date.

### Estimates of kidney function: outcome of interest

Three methods were used to calculate estimated kidney function: the Cockcroft-Gault equation [3], abbreviated MDRD Study equation [1] and CKD-EPI formula [2] as outlined below.

$$\begin{array}{l} \mathrm{Cockcroft}‒\mathrm{Gault}\;\mathrm{creatinine}\;\mathrm{clearance}\left(\mathrm{mL}/\min\right)\\ \;=\frac{\left(140-\mathrm{age}\right)*\left[\mathrm{lean}\;\mathrm{body}\;\mathrm{weight}\;\left(\mathrm{kg}\right)\right]*C}{\left[\mathrm{serum}\;\mathrm{creatinine}\;\left(\mathrm{\mu mol}/L\right)\right]} \end{array}$$

(*C* = 1.23 for males, 1.04 for females)

$$\begin{array}{l} \mathrm{Abbreviated}\;\mathrm{MDRD}\;\mathrm{eGFR}\;\left(\mathrm{mL}/\min/1.73m^{2}\right)\\ \;=175*\left[\mathrm{serum}\;\mathrm{creatinine}\left(\mathrm{\mu mol}/L\right)/88.4\right]{}^{‒1.154}\\ \;*{\left(\mathrm{age}\right)}^{‒0.203}*\left(0.742\;\mathrm{if}\;\mathrm{female}\right)\\ \;*\left(1.212\;\mathrm{if}\;\mathrm{African}‒\mathrm{American}\right) \end{array}$$

$$\begin{array}{l} \mathrm{CKD}-\mathrm{EPI}\;\mathrm{eGFR}\left(\mathrm{mL}/\min/1.73\;m^{2}\right)=141\\ \;*\min{\left[\left(\mathrm{SCr}\;\left(\mathrm{\mu mol}/L\right)/88.4\right)/\kappa ,1\right]}^{\alpha }\\ \;*\max{\left[\left(\mathrm{SCr}\;\left(\mathrm{\mu mol}/L\right)/88.4\right)/\kappa ,1\right]}^{-1.209}\\ \;*0.993^{\mathrm{age}}*\left(1.018\;\mathrm{if}\;\mathrm{female}\right)\\ \;*\left(1.159\;\mathrm{if}\;\mathrm{African}-\mathrm{American}\right) \end{array}$$

κ = 0.7 if female, 0.9 if male

α = −0.329 if female, −0.411 if male

min = the minimum of SCr/κ or 1

max = the maximum of SCr/κ or 1

CKD stage was identified from the calculated eGFR as defined by NKF Kidney Disease Outcome Quality Initiative (KDOQI) Clinical Practices for CKD [11]. Subjects were categorized as follows: stage 1–2 = eGFR ≥60 mL/min/1.73 m^2^, stage 3 = eGFR 30–59 mL/min/1.73 m^2^, stage 4 = eGFR 15–29 mL/min/1.73 m^2^, stage 5 = eGFR <15 mL/min/1.73 m^2^.

### Statistical analysis

Continuous variables were examined for normal distribution. Descriptive analyses of the above variables were performed and stratified by sex and race using non-parametric equality-of-medians tests (continuous variables) and Chi-square tests (categorical variables). Frequency of CKD (defined as CKD stage 3 or higher estimated by any of the equations for this study) was determined for the entire study population and separately for CKD calculated by each estimated kidney function equation. Logistic regression analyses were performed to determine the association between CKD (outcome of interest) and age, sex, weight and race (predictors of interest), and for those with CKD estimated by each equation. Statistical analysis was performed using STATA/IC 11.2 (StataCorp LP, College Station, TX).

## Results

Characteristics of the 356 eligible subjects identified from pharmacy records are shown in Table 1. Estimated creatinine clearance could not be calculated for 19 subjects due to missing serum creatinine or weight measurements, and eGFR could not be calculated for 6 subjects due to missing serum creatinine values or race/ethnicity. The study population had a median age of 69 years (interquartile range 66, 74 with range 65–89 years) and 39.4% were non-Hispanic black. The overall prevalence of CKD (defined as stage 3 or higher calculated by any of the equations) was 31.4%.

**Table 1:** Characteristics of subjects prescribed metformin

**Characteristic**	**Result**
	**N = 356**
Age, years, median (IQR)	69 (66, 74)
Sex, %	
Male	47.5
Female	52.5
Weight, kg, median (IQR)	76.9 (66, 93.7)
Race, %	
Non-Hispanic black	39.4
Other	60.6
Serum creatinine, μmol/L, median (IQR)^a^	80 (70, 97)
Creatinine clearance (Cockcroft-Gault), mL/min, median (IQR)^b^	76 (60, 94)
Estimated glomerular filtration rate, mL/min/1.73 m^2a^	
Abbreviated MDRD Study equation, median (IQR)	74 (61, 87)
CKD-EPI equation, mean ± SD (range)	76 ± 19 (21–118)
Prevalence of chronic kidney disease stage 3 or higher by any measure, N (%)	110 (31.4%)

*IQR* interquartile range (25th, 75th percentile).

*SD* standard deviation.

^a^N = 350, serum creatinine values not reported for 6 subjects.

^b^N = 337, serum creatinine, race or weight not reported for 26 subjects.

**Figure 1:**
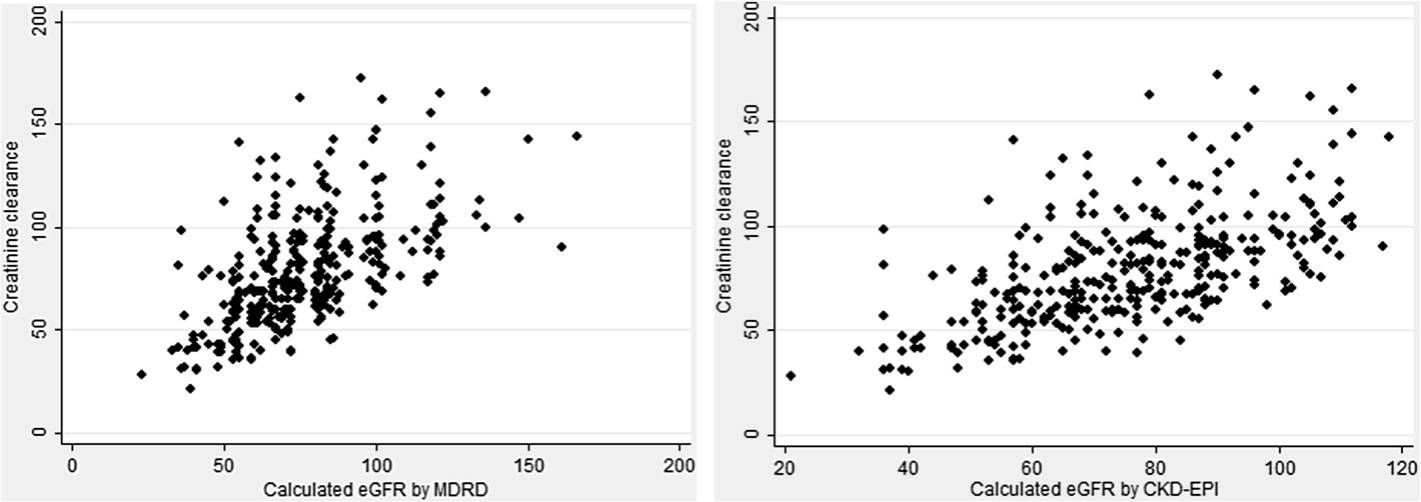
Comparison of creatinine clearance with GFR estimated by abbreviated MDRD and CKD-EPI equations.

Comparison of the estimated kidney function measurements for subjects identified as having CKD is shown in Table 2. Each equation identified more subjects as having CKD than abnormal serum creatinine alone as defined by the drug label (N = 14, 3.9%) [8,9]. The Cockcroft-Gault equation identified more subjects as having CKD (N = 80, 23.7%) compared to the abbreviated MDRD (N = 74, 21.1%) and CKD-EPI equations (N = 76, 21.7%) (*P* <0.001). The proportion of subjects categorized with CKD stages 3 and 4 were not significantly different between equations (Table 2). A small number of subjects received prescriptions for metformin despite a calculated creatinine clearance or eGFR <30 mL/min/1.73 m^2^. Median serum creatinine and eGFR were significantly lower in subjects with CKD identified by the Cockcroft-Gault equation. Subjects with CKD identified by the Cockcroft-Gault equation also were lower weight compared to those identified by the abbreviated MDRD and CKD-EPI equations. Figure 1 demonstrates both concordance and discordance between creatinine clearance and eGFR values in the study population. Discordance between calculated creatinine clearance and eGFR calculated by either the abbreviated MDRD or CKD-EPI equations was 18.9%.

**Table 2:** Comparison of subjects with chronic kidney disease (CKD) estimated by the Cockcroft-Gault, abbreviated MDRD and CKD-EPI equations

	**Cockcroft-Gault**	**Abbreviated MDRD**	**CKD-EPI**	***P*-value**
Frequency CKD stage 3 or higher, N (%)	80 (23.7%)	74 (21.1%)	76 (21.7%)	<0.001
Stage 3 (30–59 mL/min/1.73 m^2^)	78 (97.5%)	73 (98.7%)	75 (98.7%)	0.9
Stage 4 (15–29 mL/min/1.73 m^2^)	2 (2.5%)	1 (1.4%)	1 (1.3%)	0.8
Stage 5 (<15 mL/min/1.73 m^2^)	0	0	0	0.9
Serum creatinine, μmol/L, median (IQR)	97 (80, 115)	106 (88, 124)	106 (88, 124)	<0.001^c^
Estimated glomerular filtration rate, median (IQR)	47.5 (41, 55)	53 (45, 55)	52 (47, 57)	<0.001^c^

*IQR* interquartile range (25th, 75th percentile).

^c^Quantile regression to compare medians.

*MDRD* Modification of Diet in Renal Disease Study.

*CKD-EPI* Chronic Kidney Disease Epidemiology Collaboration.

Table 3 shows results of the multivariable logistic regression models. Older subjects had 13% greater likelihood of potentially inappropriate metformin prescription due to CKD defined by any equation (OR = 1.13, 95% CI 1.08-1.19, *P* < 0.001) and female subjects had 2.51-fold greater likelihood of potentially inappropriate metformin prescription due to CKD (OR = 2.51, 95% CI 1.44-4.38, *P =* 0.001). Non-Hispanic black subjects had 59% decreased odds of potentially inappropriate metformin prescription due to CKD (OR = 0.41, 95% CI 0.23-0.71, *P =* 0.001).

**Table 3:** Logistic regression models for metformin use and association with chronic kidney disease (CKD) and demographic characteristics

	**Age**		**Female**		**Weight**		**Non-Hispanic black**	
	**OR (95% CI)**	***P*-value**	**OR (95% CI)**	***P*-value**	**OR (95% CI)**	***P*-value**	**OR (95% CI)**	***P*-value**
Any CKD^d^	1.13 (1.08-1.19)	<0.001	2.51 (1.44-4.38)	0.001	0.99 (0.98-1.01)	0.32	0.41 (0.23-0.71)	0.001
CKD calculated by Cockcroft-Gault^d^	1.16 (1.10-1.23)	<0.001	1.03 (0.52-2.04)	0.94	0.92 (0.90-0.95)	<0.001	0.76 (0.39-1.50)	0.43
CKD calculated by MDRD^d^	1.09 (1.04-1.14)	0.001	3.22 (1.70-6.12)	<0.001	1.02 (1.00-1.03)	0.01	0.33 (0.17-0.62)	0.001
CKD calculated by CKD-EPI^d^	1.11 (1.05-1.16)	<0.001	3.44 (1.81-6.53)	<0.001	1.02 (1.01-1.03)	0.005	0.38 (0.20-0.71)	0.002

*MDRD* Modification of Diet in Renal Disease Study.

*CKD-EPI* Chronic Kidney Disease Epidemiology Collaboration.

CKD defined as stage 3 or higher (<60 mL/min/1.73 m^2^) by either the Cockcroft-Gault, abbreviated MDRD or CKD-EPI equations.

^d^Multivariable logistic regression model *P* < 0.001.

Comparison of CKD as calculated by each equation showed that older age was consistently associated with increased odds of potentially inappropriate metformin prescription due to CKD (Table 3). Female subjects had 3.22-fold greater likelihood of potentially inappropriate metformin prescription due to CKD calculated by the abbreviated MDRD equation (OR = 3.22, 95% CI 1.70-6.12, *P* < 0.001) and 2.44-fold greater likelihood of CKD calculated by the CKD-EPI equation (OR = 3.44, 95% CI 1.81-6.53, *P* < 0.001). Female subjects did not have significantly increased odds of potentially inappropriate metformin prescription due to CKD calculated by the Cockcroft-Gault equation (OR = 1.03, 95% CI 0.52-2.04, *P* = 0.94). Regression analyses showed significantly decreased odds of potentially inappropriate metformin prescription due to CKD for non-Hispanic blacks when the abbreviated MDRD and CKD-EPI equations were used, but not with the Cockcroft-Gault equation (Table 3).

## Discussion

CKD stage 3 or higher was common in this study population, affecting 31.4% of older adults with type 2 diabetes mellitus prescribed metformin at an urban tertiary care setting. This can neither be directly compared to previously reported estimates of CKD among all adults with diabetes mellitus, which range from 15.1% in the U.S. [21], 27.5-31% in the UK [20,22] and 38% in urban Japanese adults [23]; nor can this result be directly compared to a previous UK study which reported that 49% of adults ≥70 years old with diabetes had CKD stage 3 or higher [20], or a recent Canadian study that showed 38.7% of adults aged ≥66 years with a new metformin prescription for diabetes had CKD stage 3 or higher [24]. However, this study result may reflect kidney function of older adults with type 2 diabetes.

The Cockcroft-Gault, abbreviated MDRD and CKD-EPI equations were comparable in estimating CKD prevalence in the study population, though the Cockcroft-Gault equation identified more subjects as having CKD. Median serum creatinine was in the normal range and median eGFR was lower among subjects with CKD identified by the Cockcroft-Gault equation compared to those with CKD identified by the other equations. This demonstrates some discrepancy between estimated kidney function results using the Cockcroft-Gault equation and either the abbreviated MDRD Study equation or the CKD-EPI formula (Figure 1). This may be due to our study population consisting of older adults and racial/ethnic minorities, which were not well represented in the original populations used to derive these equations [1-3]. Additionally, some discrepancy may be due to the fact that the CKD-EPI equation is reported to be more accurate for estimating GFR >60 mL/min/1.73 m^2^[25].

The multivariable logistic regression models showed differences in the association between CKD, sex and race/ethnicity, particularly in the comparison of CKD estimated by each equation. All three equations showed that increased age was consistently associated with potentially inappropriate metformin prescription due to CKD as expected. However, the Cockcroft-Gault equation did not demonstrate statistically significant increased odds of potentially inappropriate metformin prescription due to CKD for female sex compared to the abbreviated MDRD and CKD-EPI equations. It also did not show statistically significant decreased odds of potentially inappropriate metformin prescription due to CKD for non-Hispanic blacks compared to the other equations. This is likely due to the sex and racial/ethnic differences in our study population, as the Cockcroft-Gault equation was derived mostly from Caucasian males [3]. In contrast, the abbreviated MDRD and CKD-EPI equations both showed significant associations between CKD, female sex and non-Hispanic black race.

It is worth noting that the Cockcroft-Gault equation is generally used to adjust medication doses for impaired kidney function and creatinine clearance is used in drug labeling. Clinical pharmacists primarily use the Cockcroft-Gault equation rather than the MDRD or CKD-EPI equations to estimate kidney function according to pharmaceutical manufacturer recommendations; a small survey of U.S. clinical pharmacists showed that nearly all used the Cockcroft-Gault equation to adjust dosing in kidney impairment and use of the abbreviated MDRD equation varied [4]. However, prescribing clinicians may be more likely to use eGFR (calculated from either the abbreviated MDRD equation or CKD-EPI formula) automatically reported by laboratories to evaluate kidney function, because creatinine clearance must be calculated.

It is questionable whether these eGFR discrepancies would significantly impact clinical practice in prescribing metformin. Changes in kidney function would affect a prescribing clinician’s decision to use metformin, or possibly decreasing the metformin dose due to decreasing kidney function. However, these results may indicate that using eGFR calculated from the Cockcroft-Gault equation may lead clinicians to discontinue metformin earlier than using eGFR calculated from either the abbreviated MDRD or CKD-EPI equations. It may be worthwhile to consider whether larger studies would help determine if a different CKD threshold could be used to safely prescribe metformin to older adults given the rarity of serious adverse events. Metformin-associated lactic acidosis is infrequently reported: a 2010 Cochrane review did not identify any cases of lactic acidosis from pooled data including 70,490 patient-years of metformin use, and estimated the incidence of lactic acidosis as 4.3 cases per 100,000 patient-years [10]. A recent observational study reported no increased risk of metformin-associated lactic acidosis among 51,675 Swedish adults with type 2 diabetes and kidney dysfunction [26]. Bodmer et al. reported 6 cases of lactic acidosis in a nested case–control analysis of UK subjects with type 2 diabetes taking oral medications; the estimated crude incidence rate was 3.3 cases per 100,000 patient-years [27]. Kamber et al. reported 5 cases of lactic acidosis in the Fremantle Diabetes Study group with an estimated incidence of 57/100,000 patient-years [28]. A study of U.S. adults with type 2 diabetes and CKD stage 3 nephropathy (N = 237) did not report any cases of lactic acidosis [29]. Thus, it may be inferred that the majority of older adults with diabetes and kidney impairment continue to use metformin safely.

Potential advantages for using metformin in older adults include that it is usually well-tolerated; GI adverse effects are typically dose-related and may be reduced with concurrent food intake. Metformin has a low likelihood of causing hypoglycemia as a single-drug treatment and causes less weight gain, both of which may be advantages for use in older adults. Its use may potentially delay the need for insulin, which may be difficult for older adults to administer and impact ability to live independently. Risk of metformin-associated lactic acidosis in this population may be reduced by routinely discontinuing it for anticipated changes in kidney function during acute illness and surgical procedures. Additional larger studies would be helpful to assess the benefits and risks of using metformin in older adults with type 2 diabetes.

This study has several potential limitations of note. A single serum creatinine value was used to calculate creatinine clearance and eGFR, as some subjects only had one serum creatinine value during the study period; 6 subjects had no recorded serum creatinine during the study period. This may have led to over-estimation of CKD, as we were unable to determine whether serum creatinine values were stable for ≥3 months. However, prescribing clinicians and pharmacists are more likely to use the most current serum creatinine value to guide clinical decision-making. Another limitation is that the study sample may not be representative of the general population in the United States with a greater number of Asian and Pacific Islanders who live in the Seattle area. This may under-estimate the prevalence of CKD as calculated by the MDRD and CKD-EPI equations. Lactic acidosis was not evaluated as an outcome due to difficulty identifying true metformin-associated lactic acidosis in a retrospective electronic medical record review as well as the rare frequency of metformin-associated lactic acidosis.

Despite the study’s limitations, we found a high CKD prevalence in older adults aged ≥65 years who were prescribed metformin for type 2 diabetes. CKD prevalence differed by the estimating equation used, but metformin was potentially inappropriately prescribed in a large proportion of this study population. The discrepancy between estimated kidney function calculated by the Cockcroft-Gault equation, the abbreviated MDRD Study equation and the CKD-EPI equation highlights the need for better methods to easily and accurately evaluate kidney function in the clinical practice setting with results readily available to all providers, including pharmacists. Prescribing clinicians and pharmacists may use different equations to estimate kidney function, which affects prescribing practices and evaluation of medication safety. Systems changes may also be useful to help clinicians and pharmacists determine when it is appropriate to prescribe metformin for those with CKD, especially in older adults. Possible future studies would include a longitudinal assessment of this population’s kidney function and lactic acidosis events, as well as comparing kidney function measurements with older adults taking other diabetes medications.

## Conclusions

The estimated prevalence of CKD was 31.4% in this study population of older adults prescribed metformin for type 2 diabetes mellitus, which raises the possibility of potentially inappropriate metformin use. The Cockcroft-Gault equation identified more subjects as having CKD than the abbreviated MDRD or CKD-EPI equations. This discrepancy emphasizes the need for an accurate and readily available method to easily determine kidney function of older adults in clinical practice settings. These differences in estimated kidney function may lead to disagreements between prescribing clinicians and clinical pharmacists in determining safety of metformin use.

## Competing interests

The authors declare that they have no competing interests.

## Authors’ contributions

DLH had full access to all of the data in the study and takes responsibility for the integrity of the data and the accuracy of the data analysis. DLH conceived and designed the study, collected data, analyzed and interpreted data, drafted the manuscript, and critically revised the manuscript for important intellectual content. IBA conceived and designed the study, and critically revised the manuscript for important intellectual content. BAY analyzed and interpreted data, and critically revised the manuscript for important intellectual content. All authors read and approved the final manuscript.

## Authors’ information

DLH was previously affiliated with the Division of Gerontology and Geriatric Medicine, University of Washington, Seattle, Washington, USA.

## Pre-publication history

The pre-publication history for this paper can be accessed here:

http://www.biomedcentral.com/1471-2369/15/86/prepub
